# Fathers in the spotlight: parental burden and the effectiveness of a parental skills training for anorexia nervosa in mother–father dyads

**DOI:** 10.1007/s40519-023-01597-6

**Published:** 2023-08-01

**Authors:** Michael Zeiler, Julia Philipp, Stefanie Truttmann, Tanja Wittek, Konstantin Kopp, Gabriele Schöfbeck, Dunja Mairhofer, Ellen Auer-Welsbach, Eva Staab, Andreas Karwautz, Gudrun Wagner

**Affiliations:** 1 Eating Disorder Unit, Department for Child and Adolescent Psychiatry, Medical University of Vienna, Waehringer Guertel 18-20, 1090 Vienna, Austria; 2 Department for Neurology and Psychiatry of Children and Adolescents, Klagenfurt am Wörthersee, Austria

**Keywords:** Eating disorders, Caregivers, Parental intervention, Caregiver skills, Sex differences, High-expressed emotion

## Abstract

**Purpose:**

Research on the engagement of fathers in the treatment of childhood psychiatric disorders is scarce. This study aims to investigate differences between mothers and fathers of adolescents with anorexia nervosa regarding parental burden and effectiveness of a parental skills training.

**Methods:**

Ninety-one mother–father dyads caring for a child with anorexia nervosa participated in an 8-week parental skills training and completed a set of questionnaires assessing parental psychopathology, eating disorder related burden, caregiver skills and expressed emotion at baseline and post-intervention.

**Results:**

Fathers showed lower levels of general psychological distress, depression, anxiety and eating disorder related burden as well as lower emotional overinvolvement compared to mothers. The skills training was effective in reducing parental psychopathology, eating disorder-related burden and emotional overinvolvement as well as in increasing caregiver skills with no differences between mothers and fathers. However, session adherence and the willingness to practice skills between the sessions were slightly lower in fathers.

**Conclusions:**

These findings show that fathers are a great resource for the child’s eating disorder treatment as they may counterbalance maternal emotional overinvolvement and over-protection. Furthermore, this is the first study demonstrating that fathers can profit from a parental skills training for anorexia nervosa in a similar way as mothers.

**Level III:**

Evidence obtained from well-designed cohort or case–control analytic studies.

## Introduction

There is no doubt that parents of children and adolescents with a psychiatric disorder often face high burden, are at risk to develop own psychopathological symptoms and may show maladaptive behavior impeding the child’s recovery. Parents of adolescent patients with eating disorders, particularly anorexia nervosa, report high levels of own depression and anxiety [1, 2], accommodating behavior (e.g., maladaptive adjustment to the child’s eating behavior) [3] and adverse communication patterns, such as high criticism and overflowing emotions, summarized as "High-expressed emotion" section [4] which might maintain anorexia nervosa symptoms.

Strong parental involvement in the treatment of adolescent anorexia nervosa, for example, in family-based treatment (FBT) [5], multi-family therapy (MFT) [6] or parental skills trainings [7] is recommended to provide parents with skills supporting their child, reduce maladaptive parental behavior and parental burden. Due to their important role for recovery, parents are also often seen as co-therapists [8]. Indeed, interventions for parents of adolescents with eating disorders have shown to reduce parental distress and improve outcomes in patients [7, 9–11]. However, the great majority of intervention research in parents is focused on mothers. For example, a systematic review on the efficacy of caregiver intervention in eating disorders [7] revealed that the number of fathers included in the studies was very low (< 10) in most of the studies. Other studies that included a larger number of fathers did not differentiate between mothers and fathers with regard to effects calculations. Similarly, in the most recent review on caregiver interventions for eating disorders including 28 studies [9], no study reported parental differences regarding intervention effectiveness. Thus, research on the benefit of caregiver interventions for fathers is extremely scarce. This may have different reasons: fathers usually spend less time caring for their child diagnosed with an eating disorder than mothers, are often absorbed in the work role and, in this regard, often hand over responsibility to care for the mentally ill child to the mother [12, 13]. Furthermore, some fathers show difficulties in conceptualizing the child’s eating disorder as an illness which may hinder them to engage in treatment appropriately [13, 14]. Moreover, even when fathers are involved in the eating disorder treatment, they are often not included in research studies as they tend to focus on ‘primary caregivers’ (defined as the parent spending most of the time with the child, mostly mothers) and thus, systematically exclude fathers [7, 9, 10].

Stronger involvement of fathers in the treatment of adolescents with eating disorders apart from FBT and their inclusion in research is needed for the following reasons: first, there is evidence that fathers show similar eating disorder related burden and criticism towards their child as mothers [12, 15]. Second, differences in caregiving behavior between mothers and fathers (e.g., over-protective behavior in the mother, avoidant behavior in the father) may lead to a fragmented approach towards the child which in turn may contribute to the maintenance of the eating disorder pointing to the importance of developing a common understanding and coordinated care strategies between the parents [13, 14]. Third, fathers of patients with eating disorders tend to report lower levels of anxiety, depression, stress and emotional overinvolvement [12, 16, 17] as well as similar subjective skills in coping with the child’s eating disorder [18] and less fear to engage in treatment [19] compared to mothers. Moreover, it has been reported that father’s perception of family functioning was generally more realistic and more similar to the perception of the child [20]. Forth, particularly the involvement of fathers in the treatment was associated with a better outcome in the patients [15, 21]. Altogether, this literature underlines the great resource fathers may represent for the eating disorder treatment.

Currently, there is no study available that systematically investigated mother–father differences in the effectiveness of skills trainings for parents of children and adolescents with eating disorders. However, a case report pointed to the potential of skills trainings in fathers to gain understanding of the eating disorder and to improve father–child communication [14]. A qualitative study described the fathers’ change in the understanding of their parental role in the course of FBT from low involvement in the child’s care to the realization that parental collaborative care is important for the child’s recovery [22]. Few quantitative studies in parents involved in FBT or MFT showed that the reduction in high expressed emotion and eating disorder-related burden during the course of treatment was similar for mothers and fathers [15, 23, 24]. Moreover, no differences between mothers and fathers were observed regarding satisfaction with FBT [25], while FBT session adherence was lower in fathers compared to mothers [21]. However, in view of the importance of the fathers‘ involvement in the eating disorder treatment and the limited literature on the efficacy and acceptability of parental interventions in fathers, more studies are needed investigating potential parental differences.

Considering the lack of research in fathers, in the present paper we aim to analyze the differences between mothers and fathers regarding the level of parental psychopathological symptoms, eating-disorder-related burden, expressed emotion and the perceived skills level in caring for a child with anorexia nervosa. Furthermore, we aimed to investigate parental differences regarding the pre–post-effectiveness of SUCCEAT (Supporting Carers of Children and Adolescents with Eating disorders in Austria), a skills training intervention for parents of adolescents with anorexia nervosa implemented in the clinical routine at two treatment centers specialized in eating disorders in Austria. Moreover, we explored differences between mothers and fathers regarding session adherence and adherence to different intervention components as well as regarding satisfaction and acceptability with the training. The overall aim is to find out whether fathers can benefit from SUCCEAT in the same way as mothers or whether the skills training needs to be adapted for fathers in any way.

## Methods

### Participants and recruitment

This study used baseline and post-intervention data from routine evaluations of the SUCCEAT skills training (January 2019–July 2022) implemented in the clinical routine in two centers specialized in eating disorders in Austria (Eating Disorders Unit of the Department of Child and Adolescent Psychiatry, Medical University of Vienna, 84% of the sample; and Department for Neurology and Psychiatry of Children and Adolescents, Clinic Klagenfurt am Wörthersee, 16% of the sample). We did not find systematic differences between parents recruited from the two centers with regard to sociodemographic characteristics and baseline levels of outcome variables; thus, we reported cross-center analyses only. Parents of adolescents in inpatient or outpatient treatment for anorexia nervosa are invited to participate in the 8-week skills training SUCCEAT (see below for details), while the effectiveness of the intervention is evaluated using standardized questionnaires given to the parents prior to the start of the skills training (baseline) and directly after the intervention (approximately after 3 months). In general, both parents (mothers and fathers) are motivated to participate in the training. In contrast to previous evaluation studies of SUCCEAT [10, 26], both parents were also asked to complete the evaluation assessments. Inclusion criteria were (a) parent willing to participate in the SUCCEAT training, (b) child currently in inpatient or outpatient treatment for anorexia nervosa, (c) sufficient skills of German language to follow the SUCCEAT workshop content. For the purpose of the present study, only mother–father dyads were included. Families from which only the mother or father participated in the SUCCEAT training were excluded. Parental written informed consent was obtained prior to their inclusion in the study. Ethical approval was obtained from the Ethics Commission of the Medical University of Vienna (#1840/2013).

### Intervention (SUCCEAT)

SUCCEAT is an eight-session skills training for parents originally developed by the Maudsley Hospital/King’s College London and was translated into German and adapted for parents of adolescent patients with anorexia nervosa. The intervention is based on the cognitive interpersonal maintenance model of eating disorders [27, 28] and incorporates principles of Motivational Interviewing [29]. It aims to increase parental well-being and to reduce parental burden, to provide parents with skills on how to support their child on the way towards recovery and to change intra-familiar communication patterns which may help to reduce the risk of relapse. SUCCEAT includes psychoeducational elements (e.g., etiology of anorexia nervosa and consequences of the disorder for the brain and behavior), reflection of caregiving behavior (e.g., extent of controlling behavior and emotional involvement) based on animal metaphors, a training on Motivational Interviewing skills to facilitate behavioral change in the patients, discussion of supportive parental behavior depending on the child’s motivation for change, and discussion of specific problematic behavior (e.g., vomiting, excessive exercising). Parents are advised to practice newly learned skills between the weekly sessions (called “experiments”). In addition, parents receive session handouts and are advised to read the book by Treasure et al. [30] in which further experiments are suggested. The SUCCEAT training is facilitated by two coaches (usually a psychologist and a child and adolescent psychiatrist) experienced in eating disorders who have received a training in conducting the intervention and a training in Motivation Interviewing techniques. The skills training was offered in one of three formats: (a) as face-to-face group workshops (parents of approximately eight patients each), (b) as group workshops conducted via videoconference (since the onset of COVID-19 pandemic) and (c) as online modules for self-study with weekly contact with and feedback from one of the coaches (this format was only offered until mid of 2019). The parents were not able to choose the format themselves; rather, the delivery format was predetermined by the study team considering the availability of coaches and physical distancing measures during the COVID-19 pandemic. In this study, we did not differentiate between the formats of SUCCEAT as we previously showed that the formats yielded quite similar intervention effects (Truttmann et al., 2020; SUCCEAT Team, unpublished). Moreover, mothers and fathers within a parental dyad received the same intervention format. More details on the SUCCEAT intervention content were published by Franta et al. [31].

### Measures and instruments

The following instruments were obtained in parents prior to (baseline) and after (post-intervention) the SUCCEAT skills training using an online questionnaire [32].

#### General psychopathological distress and parental psychopathology

The General Health Questionnaire, GHQ [33] was used to assess general psychological distress (possible score range: 0–12, *α* = 0.91). The level of depression was obtained with the Beck Depression Inventory, BDI [34] (possible score range: 0–63, *α* = 0.90) with scores ≥ 14 indicating a clinically relevant depression. The State/Trait Anxiety Inventory, STAI [35] was used to obtain the current level of anxiety (state score, *α* = 0.90) and the general level of anxiety (trait score, *α* = 0.90).

#### Eating disorder related burden

Eating disorder related burden was measured with the Eating Disorder Symptom Impact Scale, EDSIS [36, 37] which assesses the parental burden related to the child’s eating disorder (mealtime difficulties, illness awareness, feelings of guilt, social isolation, burden related to the child’s confrontational and binge-purging behavior). The total score (*α* = 0.86; higher scores indicating higher general eating disorder related burden) and subscale scores (0.70 ≤ *α* ≤ 0.86) indicating burden in specific areas were used in the present study.

#### Caregiver skills

The subjective level of caregiver skills was assessed with the Caregiver Skills Scale, CASK [38, 39]. Different skills relevant for parents caring for a child with an eating disorder (including emotional intelligence, frustration tolerance, bigger picture thinking, avoidance of nagging conversations—‘Biting-tongue’, accepting the eating disorder as an illness, self-care behavior) are rated on a 0–100 scale (100 = best possible skills level). Mean scores were calculated (range: 0–100) with the total score (α = 0.94) representing the overall subjective skills level and the subscale scores (0.73 ≤ *α* ≤ 0.85) representing the skills level in specific areas.

#### High-expressed emotion

The Family Questionnaire, FQ [40] was used to obtain the level of expressed emotion including the level of criticism, CC (*α* = 0.92) and emotional overinvolvement, EOI (*α* = 0.79). Cutoff values of 23 (CC) and 27 (EOI) proposed by Wiedemann et al. [40] were used to define high-expressed emotion.

### Adherence to and satisfaction with the intervention

Intervention adherence was obtained via records of session participation and by single items assessed post intervention regarding (1) their (subjective) level of active participation during the training sessions, (2) the amount of performed “experiments” between the sessions recommended by the coaches, (3) the amount of manual/book read as well as the amount of performed “experiments” suggestion in the manual/book. These items were rated on a 5-point Likert scale (1 = not at all/none, 5 = very often/all). Furthermore, satisfaction with the SUCCEAT intervention was assessed using single items regarding (1) general satisfaction with SUCCEAT, (2) how helpful SUCCEAT was in general, (3) the extent to which the content was implementable in contact with the child, subsequently called “practicability”, (4) the comprehensibility of the coaches’ suggestions and (5) the extent to which the intervention was stress-relieving. These items were rated on a 5-point Likert scale (1 = very low/bad, 5 = very high/good).

### Statistical analysis

The statistical analysis was performed with IBM SPSS Statistics 27.0. First, we compared the baseline level of psychological burden, eating disorder-related burden, subjective skills level and expressed emotion between mothers and fathers using *t* tests for matched samples and calculating Cohen’s *d* effect sizes for the difference between the parents. Bonferroni-adjusted significance levels were used to account for multiple comparisons. We repeated these analyses using general linear models including the time of parental contact during weekdays as covariate. Furthermore, we calculated the percentage of clinically relevant depression scores as well as the percentage of high criticism and high emotional overinvolvement and compared mothers and fathers using McNemar tests. Second, differences between mothers and fathers regarding the SUCCEAT intervention effects (pre–post change) were analyzed using general linear mixed models (main effects of time and parent, time x parent interaction effect; controlled for the SUCCEAT delivery format). Again, we used Bonferroni-corrected significance levels to account for multiple testing. Third, we investigated mother–father differences regarding intervention adherence and satisfaction using a *t* test for matched samples (number of workshop sessions completed) and Wilcoxon signed rank tests (single items measuring adherence to different intervention components and satisfaction).

## Results

### Sample characteristics

The sample consisted of 91 mother–father dyads who have participated in the study between January 2019 and December 2022. The majority of data were obtained during the COVID-19 pandemic period (70.3%), while the remaining data were obtained prior to the start of the pandemic (29.7%). Most parental dyads were married and lived in the same household (89%). The main characteristics of the mother–father dyads are shown in Table 1. Fathers were comparable to mothers with regard to age, educational level, nationality, employment status and the presence of a lifetime psychiatric disorder. However, fathers had more weekly working hours compared to mothers. A higher percentage of mothers was affected by an own lifetime eating disorder and mothers had more contact to the child with the eating disorder during weekdays compared to fathers. The majority of mother–father dyads participated in the videoconference format of SUCCEAT (69.2%), 15.4% received the face-to-face workshop format and 15.4% the online modules format.

**Table 1:** Sample characteristics of 91 mother–father dyads

	Fathers (*n* = 91)	Mothers (*n* = 91)
Age (Mean, SD)	49.71 (5.59)	47.64 (4.41)
Highest educational level (%)		
University degree	64.8%	65.9%
Secondary education	17.6%	20.9%
Below secondary education	17.6%	13.2%
Nationality (%)		
Austria	89.0%	87.9%
Other	11.0%	12.1%
Current employment status (%)		
Employed	96.7%	95.6%
Not employed	3.3%	4.4%
Average working hours per week (Mean, SD)	41.9 (9.45)	30.61 (8.59)
Prior support due to the child’s eating disorder (%)	23.1%	34.1%
Parental lifetime eating disorder diagnosis (%)	2.2%	14.3%
Parental lifetime psychiatric disorder (other than an eating disorder (%)	12.1%	12.1%
Contact to the child during weekdays (5)		
0–1 h/day	25.3%	6.6%
1–2 h/day	29.7%	24.2%
3–4 h/day	29.7%	23.1%
> 4 h/day	15.4%	46.2%

The adolescents diagnosed with anorexia nervosa were predominantly female (97.8%), had a mean age of 14.69 (SD: 2.10) and an eating disorder duration of 9.69 months (SD: 8.00) on average. At the timepoint of the parents’ participation in the SUCCEAT intervention, 61.5% of patients received outpatient treatment and 38.5% received inpatient treatment for anorexia nervosa.

### Parental burden and skills levels prior to the SUCCEAT intervention

At baseline, fathers showed significantly lower levels of general psychological distress, depression, actual level of anxiety, eating disorder related burden and emotional overinvolvement compared to mothers, while these effects were medium-sized (see Table 2 for details). Fathers further tended to have lower scores on trait anxiety and criticism as well as higher self-rated caregiver skills compared to mothers; however, effect sizes were low and differences did not reach statistical significance on a Bonferroni-corrected significance level. We repeated these analyses using the amount of contact with the child during weekdays as potential confounding variable. Indeed, this variable neither had an impact on the outcome variables, nor changed the pattern of results presented in Table 2.

**Table 2:** Differences between fathers and mothers with regard to outcome variables measured at baseline

	Mean (SD)		Test statistic		Effect size
	Fathers	Mothers	*t* (df)	*p*	Cohen’s *d*
GHQ	3.93 (3.32)	5.98 (3.58)	− 4.610 (90)	** < 0.001**	− 0.48
BDI	8.36 (7.17)	13.49 (7.74)	− 4.933 (89)	** < 0.001**	− 0.52
STAI State	41.81 (10.34)	48.33 (11.89)	− 4.437 (89)	** < 0.001**	− 0.47
STAI Trait	37.22 (10.10)	41.02 (9.76)	− 2.567 (89)	0.012	− 0.27
EDSIS Total	29.19 (10.75)	35.49 (12.30)	− 4.374 (89)	** < 0.001**	− 0.46
CASK Total	73.41 (14.36)	68.98 (14.87)	2.371 (89)	0.020	0.25
FQ CC	20.26 (5.38)	21.52 (5.43)	− 2.158 (89)	0.034	− 0.23
FQ EOI	22.91 (3.88)	26.60 (4.90)	− 5.792 (89)	** < 0.001**	− 0.61

Bold values indicate statistically significant differences on a Bonferroni-adjusted significance level of 0.006. *GHQ* General Health Questionnaire, *BDI* Beck Depression Inventory, *STAI* State Trait Anxiety Inventory, *EDSIS* Eating Disorder Symptom Impact Scale, CASK Caregiver Skills Scale, *FQ CC* Family Questionnaire Criticism Score, *FQ EOI* Family Questionnaire Emotional Overinvolvement Score

Looking at the EDSIS subdomains, we observed that mothers showed higher scores in the ‘mealtime difficulties’ (*p* = 0.001), ‘illness awareness’ (*p* < 0.001) and ‘guilt’ (*p* = 0.003) subscales compared to fathers. CASK subscale analyses yielded significantly higher scores in the ‘self-care’ (*p* < 0.001) and ‘acceptance’ (*p* < 0.001) subscales for fathers compared to mothers.

With regard to the established cutoff in the BDI questionnaire, a significantly lower percentage of fathers (18.7%) compared to mothers (44.4%) showed clinically relevant depression scores (McNemar: *p* = 0.004). Regarding high-expressed emotion, a lower percentage of fathers (25.3% vs. 47.8% in mothers) showed elevated emotional overinvolvement (McNemar: *p* < 0.001), while there was no difference regarding the percentage of elevated criticism scores (fathers: 26.4% vs. mothers: 36.7%, McNemar: *p* = 0.286).

### Differences between fathers and mothers regarding the SUCCEAT intervention effects

The following results are based on a sample of 70 mother–father dyads who have completed both, baseline and post-intervention assessments. The GLMM models revealed a statistically significant effect of time for the majority of outcome variables (except for the STAI trait and FQ CC scores) indicating a baseline-to-post-intervention reduction in parental psychopathology, burden and emotional overinvolvement as well as an increase in caregiver skills. Noteworthy, there were no time *x* parent interaction effects for any of the outcome variables indicating that the improvements in outcome scores are independent from the caregiver’s sex (see Table 3). The statistically significant main effects of the parent found for most of the outcome scores reflected the mother–father differences observed at baseline (Table 2).

**Table 3:** Results of general linear mixed models (GLMM) investigating changes in outcome variables for mother and fathers

	Fathers (Mean, SD)		Mothers (Mean, SD)		GLMM effects (*F*, df_1_ = 1, df_2_ = 69, *p* value)^a^		
	*Baseline*	*Post*	*Baseline*	*Post*	Time	Parent	Time *x* parent
GHQ	3.94 (3.33)	1.94 (3.10)	5.96 (3.51)	3.54 (3.67)	*F* = 26.756, ***p* < 0.001**	*F* = 15.908, ***p* < 0.001**	*F* = 0.182, *p* = 0.671
BDI	7.87 (6.62)	5.30 (5.77)	13.22 (7.87)	9.04 (8.14)	*F* = 18.097, ***p* < 0.001**	*F* = 19.432, ***p* < 0.001**	*F* = 0.478, *p* = 0.492
STAI State	40.74 (10.08)	37.93 (10.25)	48.14 (11.78)	43.68 (12.12)	*F* = 11.232, ***p* = 0.001**	*F* = 16.389, ***p* < 0.001**	*F* = 0.005, *p* = 0.943
STAI Trait	35.88 (9.62)	35.26 (9.97)	41.36 (9.70)	40.54 (10.50)	*F* = 1.719, *p* = 0.194	*F* = 10.347, ***p* = 0.002**	*F* = 0.342, *p* = 0.560
EDSIS Total	29.38 (9.88)	25.50 (11.66)	35.76 (12.44)	29.25 (13.38)	*F* = 17.114, ***p* < 0.001**	*F* = 19.046, ***p* < 0.001**	*F* = 0.521, *p* = 0.473
CASK Total	74.20 (13.85)	79.71 (12.65)	69.25 (14.96)	76.04 (14.42)	*F* = 12.491, ***p* < 0.001**	*F* = 4.776, *p* = 0.032	*F* = 0.009, *p* = 0.924
FQ CC	20.59 (5.34)	20.12 (4.87)	22.20 (5.53)	20.49 (5.09)	*F* = 4.632, *p* = 0.035	*F* = 3.492, *p* = 0.066	*F* = 3.898, *p* = 0.052
FQ EOI	22.87 (3.89)	21.80 (4.17)	26.86 (4.90)	24.93 (5.30)	*F* = 16.306, ***p* < 0.001**	*F* = 24.200, ***p* < 0.001**	*F* = 0.402, *p* = 0.528

Bold values indicate statistically significant differences on a Bonferroni-adjusted significance level of 0.006

*GHQ* General Health Questionnaire, *BDI* Beck Depression Inventory, *STAI* State Trait Anxiety Inventory, *EDSIS* Eating Disorder Symptom Impact Scale, *CASK* Caregiver Skills Scale, *FQ CC* Family Questionnaire Criticism Score, *FQ EOI* Family Questionnaire Emotional Overinvolvement Score

^a^Effects were corrected for the SUCCEAT delivery format (videoconference format vs. other format) which was used as a covariate in the statistical model

### Parental differences regarding intervention adherence and satisfaction

**Fig. 1:**
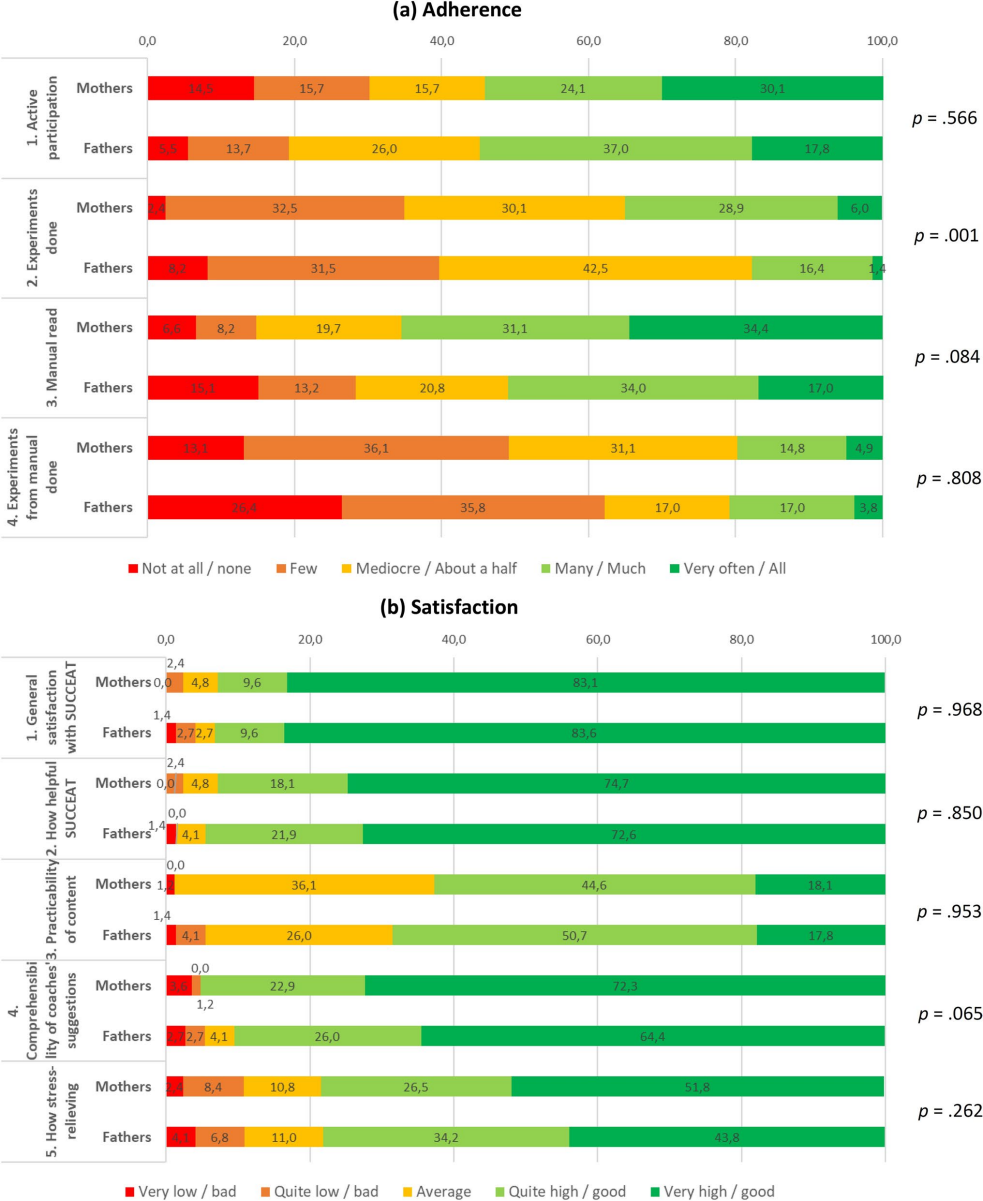
Differences between mothers and father regarding **a** intervention adherence and **b** satisfaction with the SUCCEAT intervention

The average number of completed sessions was slightly but significantly lower in fathers (5.96 out of 8 sessions, SD: 2.38) compared to mothers (6.52 out of 8 sessions, SD: 1.84; *t* = 2.051, *p* = 0.043). Fathers reported having done less experiments between the workshop session compared to mothers (*p* = 0.001). No statistically significant differences between mothers and fathers were observed for other adherence variables (extent of active participation during the workshop session, amount of the manual/book read, amount of experiments done suggested by the manual/book, see Fig. 1a). With regard to acceptability and satisfaction, we did not find statistically significant differences between the parents in any of the considered variables (Fig. 1b). Noteworthy, the great majority of mothers (92.7%) and fathers (93.2%) reported to be very satisfied or quite satisfied with the SUCCEAT intervention in general.

## Discussion

This paper aimed to investigate differences in mother–father dyads regarding parental burden, caregiving behavior and skills for caring for an adolescent with anorexia nervosa as well as mother–father differences regarding the effectiveness of a parental skills training. Fathers showed lower psychological distress, psychopathology, eating disorder-related burden and emotional overinvolvement compared to mothers which was independent from the amount of time spent for caregiving. Improvements in outcome variables from baseline to the post-intervention assessment were similar for mothers and fathers. Adherence to the intervention sessions and the amount of exercises performed between the sessions were slightly lower in fathers compared to mothers, while there were no parental differences reading satisfaction and acceptability with SUCCEAT.

In line with the literature [12, 17, 24], fathers showed lower levels of adverse emotional reactions (symptoms of depression, anxiety and emotional overinvolvement) compared to mothers. Less paternal emotional involvement might also be reflected by greater self-care behavior and higher acceptance of the child’s eating disorder found in fathers in the present study. Rienecke and Richmond [41] highlighted that mothers and fathers tend to react differently to the child’s eating disorder with mothers often showing more anxious behavior and fathers often using more cognitive and avoiding strategies to cope with the illness. While overly rational behavior may be perceived by the child as rejection, less paternal emotional responses may also be advantageous. As emphasized by Treasure [13], fathers may counterbalance maternal over-protective behavior and emotional overinvolvement and thus, may be well suited to support the child through “calm encouragement” (p. 386). Indeed, the most pronounced parental difference found in the present study was the higher level of emotional overinvolvement in mothers.

In contrast to another study that found similar levels of eating disorder related burden [15], we observed higher levels of burden (including feelings of guilt and perceived difficulties during mealtimes) in mothers compared to fathers. As burden levels were previously associated with the amount of time for caregiving [12], this was not the case in the present study indicating that fathers may feel less burdened by the child’s eating disorder per se. However, general sex differences in disclosing specific negative emotions such as guilt must be taken into account when interpreting these results [42]. Lower paternal levels of burden, stress and psychopathology potentially give father more power to actively engage in the treatment which may counterbalance higher burden levels in mothers. In sum, the current study highlights the potential of fathers for the eating disorder treatment of their child and encourages greater involvement of fathers in the future.

This is the first study showing that fathers can profit from a parental skills training for adolescent anorexia nervosa in a similar way than mothers do. Indeed, the reduction in psychopathology and burden levels as well as in emotional overinvolvement and the increase in caregiver skills levels did not differ between the parents. This is line with previous research on the parental effects of FBT and MFT [15, 23] showing that both parents can similarly profit from the interventions. This study, therefore, expands the literature on the effectiveness of caregiver interventions in eating disorder that had a strong focus on mothers [7, 9]. The level of criticism towards the child, however, did not significantly decrease for both parents which is consistent with other studies showing that criticism was an outcome variable that is difficult to change and prone to rise again in the long-term [23, 24]. Thus, strengthening intervention content on communication skills to reduce critical comments towards the child’s behavior should be considered.

Although fathers tended to participate in less intervention sessions compared to mothers, the overall paternal adherence rate was high which is not self-evident considering the significantly low engagement levels of fathers in behavioral trainings for other childhood psychiatric disorders [43]. Moreover, it should be considered that the participation of mothers and fathers in a skills training at the same time might be difficult per se as other responsibilities (e.g., care for younger children) may be a hindering factor. Thus, the high adherence rates observed in both parents in the present study highlights the high general engagement level of the parents. High paternal adherence to a parental intervention may not only be important for reducing their burden associated with the child’s mental health disorder but also indirectly as outlined by Hughes et al. [21]. This study discussed that high engagement of fathers may signal to the adolescents that they care about them and that they are committed to their recovery. Moreover, also mothers may feel better supported in their caregiver role which may be beneficial for family cohesion in general. The fact that there was no difference between mothers and fathers regarding the extent to which they actively participated during the workshop sessions indicates that fathers have not just been passive listeners handing over the main responsibility to the mothers as previously regarded as a main challenge [22]. However, the findings from this study indicate that fathers, a bit more than mothers, need to be motivated to practice the newly learned skills with the child. To reach the maximum impact of SUCCEAT, this should be considered in future parental trainings. Finally, we found very high satisfaction with SUCCEAT for both parents. Particularly for fathers, this finding is promising as previous research has pointed to paternal difficulties in expressing emotions or reflecting about own emotional reactions to the child’s eating disorder [22] which are key elements of SUCCEAT. This underlies the usefulness of the SUCCEAT skills training for both parents.

### Strengths and limitations

Strengths of this study are the inclusion of a relatively large sample of parents with anorexia nervosa and the focus on mother–father dyads which allows a direct comparison between mothers and fathers independent from the child’s symptomatology, the timepoint of inclusion in the study and the intervention format. However, this study also has the following limitations. We do not have data from parents who did not participate in the skills training; thus, particularly the sample of fathers might be restricted to those who were motivated to learn more about the child’s eating disorder and to change their caregiving behavior. Moreover, we only obtained parental burden, caregiver skills and behavior from a subjective level. Thus, general sex differences in expressing psychopathological symptoms and burden might have influenced the results. More objective measures of caregiver skills and behavior, for example, by behavioral observation techniques, may provide additional insight to mother–father differences. Furthermore, we only obtained the short-term effectiveness of SUCCEAT in this study. Therefore, we do not know whether the improvements observed in fathers are sustained as previously shown in a sample of primary caregivers (mostly mothers) [10]. Finally, in the present study we neither obtained the symptomatology of the adolescents with anorexia nervosa, nor its change over the course of treatment. Thus, we cannot answer the question whether the involvement of both parents in the skills training is beneficial for the child’s recovery. SUCCEAT participation of (primarily) mothers has been previously linked to improvements in the child’s eating disorder outcome [32]; however, the specific role of fathers for improvements in the psychopathology of the patients’ needs to be further investigated. Future studies may, for example, focus on how mothers’ and fathers’ involvement in the treatment change their attitudes, insight and behavior regarding the eating disorder and how this, in turn, affects the child’s treatment course.

### What is already known on this subject?

Mothers often take on the main responsibility of caring for a child with anorexia nervosa and experience high level of psychological distress. Previous research focusing on mothers has demonstrated high effectiveness of parental skills trainings in reducing burden and improving caregiver skills.

### What this study adds?

This study showed that fathers of adolescent patients with anorexia nervosa showed less psychological distress, eating disorder related burden and emotional overinvolvement compared to mothers. It is also one of the fist studies demonstrating that fathers can similarly profit from a specialized parental skills training as mothers. A greater involvement of fathers in the adolescent eating disorder treatment is thus highly encouraged.

## Conclusion

To conclude, due to lower levels of emotional overinvolvement, anxiety and depression, fathers are a great resource for the collaborative care in adolescent anorexia nervosa. The present study indicates that fathers can profit from a parental skills training in a similar way as mothers. Thus, they should be involved in the child’s treatment to a greater extent to reach a joint parental understanding of the disorder and how to support the child best on his/her way towards recovery. A greater involvement of fathers may also relieve the burden of mothers who are often under a great deal of strain. Fathers should be motivated to participate in as many intervention sessions as possible and to practice the newly learned skills with the child between the sessions. However, parental differences regarding intervention adherence were small and might not be practically relevant. The present results may also be inspiring for the research on the inclusion of fathers in the treatment of other psychiatric disorders in childhood and adolescence. Burden and suffering of parents, including fathers, when caring for a child with a mental illness might be enormous. Providing professional parental support through structured trainings for both, mothers and fathers, may induce “*precious moments with the child*” and enables parents to “*bring new hope to the affected child*” (quotes from a father having participated in the SUCCEAT intervention).

## Data Availability

The data related to this study are available from the corresponding author upon reasonable request.
